# Prospective evaluation of the effectiveness of combined treatment of macular edema secondary to retinal vein occlusion with intravitreal bevacizumab and dexamethasone implants

**DOI:** 10.5935/0004-2749.20230040

**Published:** 2023

**Authors:** María Moreno-López, Pablo de-Arriba-Palomero, Fernando de-Arriba-Palomero, Federico Peralta Ituruburu, Elisabet de Dompablo, Francisco José Muñoz-Negrete

**Affiliations:** 1 Ophthalmology Service, Hospital Universitario Ramón y Cajal, IRYCIS, Madrid, Spain.

**Keywords:** Retinal vein occlusion/complications, Macular edema/drug therapy, Angiogenesis inhibitors/ therapeutic use, Dexamethasone/ administration & dosage, Intravitreal Injections, Bevacizumab, Tomography, optical coherence, Visual acuity, Oclusão da veia retiniana/complicações, Edema macular/tratamento farmacológico, Inibidores de angiogênese/uso terapêutico, Dexametasona/administração & dosagem, Injeções intravítreas, Bevacizumab, Tomografia de coerência óptica, Acuidade visual

## Abstract

**Purpose:**

To evaluate the effectiveness of intravitreal bevacizumab injections following a single dexamethasone implant in the treatment of macular edema secondary to branch and central retinal vein occlusion.

**Methods:**

This was a prospective interventional non-comparative study, 44 eyes of patients with naïve macular edema related to branch and central retinal vein occlusion were treated with a dexamethasone implant. Patients were followed-up at four-week intervals from the second to the sixth month. If persistent or recurrent macular edema occurred during this period, the patient was treated with intravitreal bevacizumab injections on an as-needed basis. The outcome measures were best-corrected visual acuity and central macular thickness changes.

**Results:**

The mean best-corrected visual acuity changed from 0.97 ± 0.33 LogMAR at baseline to 0.54 ± 0.40 at the six-month post-implant examination (p<0.00001). Improvement ≥3 Snellen lines were seen in 20 eyes (45.54%). The mean central macular thickness at baseline was 670.25 ± 209.9 microns. This had decreased to 317.43 ± 112.68 microns at the six-month follow-up (p<0.00001). The mean number of intravitreal bevacizumab injections received in the six months post-implant was 2.32. The mean time from dexamethasone implant to first anti-VEGF injection was 3.45 months.

**Conclusions:**

Intravitreal bevacizumab injections following a single dexamethasone implant were found to improve best-corrected visual acuity and central macular thickness in patients with macular edema due to branch and central retinal vein occlusion at six months, with few intravitreal injections required.

## Abstract (pt)

**Objetivo:**

Avaliar a eficácia da combinação de injeções intravítreas de bevacizumabe em olhos com edema macular secundário à oclusão de ramo e da veia central da retina após um único implante de dexametasona.

**Métodos:**

Foi realizado um estudo prospectivo intervencionista não comparativo com 44 olhos de pacientes com edema macular relacionado à oclusão de ramo e veia central da retina, sem tratamento prévio e tratados com um único implante de dexametasona, que foram acompanhados em intervalos de quatro semanas do segundo ao sexto mês. Se fosse constatado edema macular persistente ou recorrente durante esse período, os pacientes eram tratados com injeções intravítreas de bevacizumabe em um regime ajustado conforme a necessidade. Foram estudadas a melhor acuidade visual corrigida e alterações da espessura macular central.

**Resultados:**

A média da melhor acuidade visual corrigida mudou de 0,97 ± 0,33 LogMAR iniciais para 0,54 ± 0,40 no exame de 6 meses (p<0,00001). Vinte olhos (45,54%) melhoraram 3 linhas de Snellen ou mais. A média da espessura macular central inicial foi de 670,25 ± 209,9 µm e diminuiu para 317,43 ± 112,68 µm na visita de 6 meses (p<0,00001). O número médio de injeções intravítreas de bevacizumabe em 6 meses foi de 2,32 e o tempo médio entre o implante de dexametasona e a primeira injeção de anti-VEGF foi de 3,45 meses.

**Conclusão:**

Injeções intravítreas de bevacizumabe após um único implante de dexametasona podem proporcionar um aumento da melhor acuidade visual corrigida e diminuição da espessura macular central aos 6 meses em pacientes com edema macular devido à oclusão de ramo e da veia central da retina, com poucas injeções intravítreas.

## INTRODUCTION

Among retinal vascular disorders, retinal vein occlusion (RVO) is the second most common cause of vision loss^(1-3)^. Approved treatments include anti-vascular endothelial growth factor (anti-VEGF): ranibizumab (Lucentis^®^; Genentech/Roche, USA)^(4-6)^ and aflibercept (Eylea, Regeneron Pharmaceuticals, USA; Bayer Pharma AG, Germany)^(7,8)^, and steroid implants such as the 0.7 mg dexamethasone implant (DI) (Ozurdex^®^, Allergan, USA)^(9)^. Bevacizumab (Avastin^®^; Genentech, USA and Roche, Germany) is an alternative anti-VEGF that is also used off-label to treat macular edema (ME)^(10-12)^. DI and anti-VEGF therapy have been approved for several retinal diseases and are the first-line treatment for disorders such as diabetic retinopathy^(13)^ and age-related macular degeneration^(14)^.

A drawback of anti-VEGF therapy is the need for multiple injections, especially during the first year, with some patients achieving incomplete resolution despite extensive treatment. Moreover, research suggests that achieving clinically significant gains in visual acuity takes longer with anti-VEGF than DI treatment, with averages ranging from 4 to 5.9 months versus 7 days to 2 months, respectively^(15)^. DI provides sustained release of the drug for six months, with maximum effects at two months^(9,16,17)^. The Global Evaluation of Implantable Dexamethasone in Retinal Vein Occlusion with Macular Edema (GENEVA) study group recommends DI injections every six months^(16)^. However, more recent research suggests that earlier repeated DI injections produce better results^(18)^. Although treatment with intraocular steroids carries risks of glaucoma and cataracts^(9,10)^, the side-effect profile from a single DI is favorable^(9,19,20)^.

Several studies have evaluated combined anti-VEGF and DI treatment for synergistic effects that would reduce the number of injections required and minimize side effects^(18,21-25)^. To date, however, there is no consensus on the best drug combination or therapeutic regimen.

We conducted a prospective interventional study to evaluate the efficacy of bevacizumab treatment in the six months following a single DI for persistent or recurrent treatment-naïve ME secondary to RVO. We aimed to evaluate the functional and anatomical outcomes of this combined therapeutic regimen.

## METHODS

A prospective, interventionist non-randomized study was performed at our institution from 2014 to 2018. Approval was obtained from our center’s institutional review board, and the study was conducted following the principles of the Declaration of Helsinki. This study is registered on the EudraCT database and the EU Clinical Trials Register (NEudraCT 2012-000165-20). All patients were instructed on the purpose and procedure of our study and written informed consent was obtained. This research was funded by: Spanish Health Ministry, aid for independent clinical research October 2011, project EC11-136.

### Study population

Patients older than 18 years with ME secondary to central retinal vein occlusion (CRVO) or branch retinal vein occlusion (BRVO) treated with a single DI were screened for eligibility according to our protocol criteria (Table 1). Patients treated with a single DI were screened at two months and then at four-week intervals for up to six months. Those that presented with persistent or recurrent ME were recruited. Persistent ME was defined as intraretinal or subretinal fluid present at the two-month visit with incomplete retinal fluid resolution at any previous visit. Recurrent ME was defined as an increase in central macular thickness (CMT) greater than 50 µm since the previous visit or *de novo* intraretinal or subretinal fluid.

**Table 1:** Inclusion and exclusion criteria

Inclusion criteria
a) Adults aged 18 or older.
b) Diagnosis of acute ME secondary to BRVO or CRVO proven by clinical examination and OCT. Acute ME was defined as less than 12 months duration referred by the patients as sudden vision loss.
c) Naïve ME due to RVO having received a single DI within the previous 6 months.
d) BCVA better than hand motion and worse than 20/40.
e) CMT on OCT >300 mm, with intraretinal and/or subretinal fluid.
**Exclusion criteria**
a) Patients having received any oral or intraocular treatment for ME prior to DI.
b) Patients having had ocular surgery or laser treatment within the previous 4 months.
c) History of systemic conditions that prevent the use of intraocular bevacizumab (pregnancy, lactation, stroke, uncontrolled systemic hypertension, or any other uncontrolled systemic disease).
d) Eyes being treated with topical prostaglandin analogs.
e) Allergy to any components of Avastin^®^.
f) Presence of a clinically significant epiretinal membrane or vitreomacular traction on OCT.
g) Presence of diabetic retinopathy, active retinal or optic disc neovascularization, active or past history of choroidal neovascularization, presence of rubeosis iridis, any active ocular infection, aphakia or anterior-chamber intraocular lens, glaucoma or current ocular hypertension requiring more than one medication to control IOP in the study eye.
h) Patients currently using or anticipating the use of systemic steroids, or any ocular condition in the study eye that, in the opinion of the researcher, would prevent a 3 -line improvement in visual acuity.
i) Patients who have a loss of vision for any other cause.
j) Patients who were lost to follow-up during the study period.

### Treatment

Patients with persistent or recurrent ME were treated with 1.25 mg intravitreal bevacizumab injections (IVB) on an as-needed basis. All intravitreal injections were performed under aseptic conditions in the operating room. Eyelids were cleaned with 10% povidone-iodine and a drop of diluted 5% povidone-iodine was applied to the bulbar conjunctiva before and after the injection.

### Data collection

A detailed ophthalmic examination was conducted during each visit. This included slit-lamp biomicroscopy, Snellen best-corrected visual acuity (BCVA) measurement, dilated fundoscopy, Goldmann applanation tonometry, retinography, and spectral-domain optical coherence tomography (SD-OCT) macular cube 512 x 128 analysis (Cirrus HD^®^, Zeiss Göschwitzer, Germany) to measure CMT. The decimal Snellen BCVA scores were converted into logarithms of the minimum angle of resolution (logMAR) for statistical comparison^(26)^. Fluorescein angiography (FA) was performed at the baseline visit to detect ischemic versus non-ischemic RVO.

### Study objectives

The primary outcomes were BCVA improvement and decreased CMT six months after the DI. In cases with no intraretinal or subretinal fluid and a CMT <300 µm, the anatomical results were evaluated by absolute success rate. Those with a CMT reduction >30% or with presence of intraretinal or subretinal fluid at six months after the DI were evaluated by relative success rate. Secondary outcomes were the proportion of eyes that gained at least three Snellen lines and the number of IVB administered.

### Statistical analysis

We confirmed the sample’s normality with Shapiro-Wilk, histogram plot and kurtosis and employed student’s paired t-tests to evaluate changes in best-corrected visual acuity (BCVA) and CMT. Two-sample t-tests were used to evaluate differences between the samples. All tests were two-tailed, the level of significance was set at p<0.05, and 95% confidence intervals were used. The results were shown as means ± standard deviations. Statistical tests were performed using STATA statistical analysis software, version 13 (StataCorp; Texas, USA).

## RESULTS

Our study group comprised 44 eyes from 44 patients with ME following RVO who met our protocol criteria and completed the follow-up period. Table 2 summarizes the patients’ baseline characteristics. The study group’s age distribution was as follows: 40-50 years (three patients), 50-60 years (seven patients), 60-70 years (10 patients), 70-80 years (20 patients), and >80 years (four patients). Of the 44 eyes, 35 (79.54%) presented with ME onset less than three months after DI. FA was performed on 39 eyes (14 with CRVO and 25 with BRVO). Ischemic vein occlusion (IVO) was defined in CRVO as a diameter of nonperfusion greater than 10 discs, and in BRVO as a non-non-perfusion diameter greater than five discs. IVO was detected in two of the 14 CRVO cases (14.28%) and six of the 25 BRVO cases (24%).

**Table 2:** Baseline characteristics of study patients

Total cases	44
Age (years, mean ± SD)	67.91 ± 11.32 (Range 44-87)
Gender (M/F)	24 / 20 (54.55%/45.45%)
Eye (RE/LE)	25 / 19 (56.81% / 43.18%)
Duration of ME (months, mean± SD)	2.34 ± 2.54
RVO type: CRVO / BRVO. (n, proportion)	17 / 27 (38.63%/61.36%)
Fluorescein angiography	
- CRVO (n)	14
- BRVO (n)	25
Ischemic vs non- ischemic	
- CRVO (n, proportion)	- 2 ischemic/ 12 non-ischemic (14.28%/85.71%)
- BRVO (n, proportion)	- 6 ischemic/19 non-ischemic (24%/76%)

M/F= Male/Female; RE/LE= Right eye/Left eye; RVO= Retinal vein occlusion; CRVO= Central retinal vein occlusion; BRVO= Branch retinal vein occlusion.

### Visual acuity

The mean Snellen BCVA at baseline was 0.14 ± 0.12 (LogMAR 0.97 ± 0.37). The BCVA was ≤0.05 for 15 eyes (34.09%), >0.05 and ≤0.1 for nine eyes (20.45%), and >0.1 and ≤0.5 for 20 eyes (45.45%). The mean Snellen BCVA at six months was 0.39 ± 0.25 (LogMAR 0.54 ± 0.40). The final Snellen BCVA was ≤0.1 for eight eyes (18.18%), >0.1 and <0.5 for 19 eyes (43.18%), and ≥0.5 for 17 (38.63%) eyes.

The mean BCVA changed significantly from a LogMAR of 0.97 ± 0.37 at baseline to 0.54 ± 0.40 at the six-month examination (p<0.00001). Twenty eyes (45.54%) had improved by three or more Snellen lines at six months. Overall, 42 eyes (95.45%) showed the same or improved BCVA at six months, while 2 eyes (4.54%) had worsened BCVA.

Considering the type of RVO, 44.44% of the BRVO (12/27) and 47.05% (8/17) of the CRVO eyes gained three or more Snellen lines. 33.33% (9/27) of the BRVO and 47.05% (7/17) of the CRVO eyes achieved a BCVA of ≥0.5 at six months (Table 3).

**Table 3:** Functional and anatomical results after six months of combination treatment with a single dexamethasone implant followed by as-needed intravitreal bevacizumab injections in patients with macular edema secondary to retinal vein occlusion.

	Visual acuity (LogMAR ±SD)			CMT (mm ±SD)		
	Baseline	Month 6	p*	Baseline	Month 6	p*
All (n=44)	0.97 ± 0.37	0.54 ± 0.40	<0.00001	670 ± 209.9	317.43 ± 112.68	<0.00001
CRVO (n=17)	0.99 ± 0.38	0.57 ± 0.47	0.0002	729 ± 225.41	316 ± 138.53	<0.00001
BRVO (n = 27)	0.97 ± 0.37	0.52 ± 0.36	<0.0001	633.25 ± 194.72	318.11 ± 95.84	<0.00001
*p#*	0.86	0.66		0.14	0.96	

p*= paired t-test. *p*#= Two-sample paired t-test, between CRVO and BRVO.

In our study population, only 8 of the 39 cases (20.51%) studied using FA were ischemic. These ischemic cases showed improved BCVA, from a LogMAR of 1.15 ± 0.38 at baseline to a LogMAR of 0.81± 0.35 at the six-month evaluation (paired t-test, *p*=0.26). The non-ischemic cases also showed improved BCVA, from 0.96 ± 0.37 at baseline to 0.49 ± 0.43 at the six-month evaluation (paired t-test, p<0.0000). None of the ischemic eyes achieved a BCVA >0.5, and only 2 eyes (25%) gained three or more Snellen lines. Fifteen of the 31 non-ischemic eyes (48.38%) achieved a BCVA ≥0.5, and 18 (58.06%) improved by three or more Snellen lines.

### Anatomical results

The CMT of our sample decreased by a mean of 352.82 ± 236.37 µm throughout the follow-up period. The mean CMT at baseline was 670.25 ± 209.9 µm and 317.43 ± 112.68 µm at the six-month visit (p<0.00001).

Twenty-four eyes (54.54%) achieved absolute success (absence of intraretinal or subretinal fluid and a CMT <300 µm), and 33 eyes (75%) achieved relative success (a decrease in CMT >30%).

The mean baseline CMT did not differ between those eyes that achieved a BCVA gain of three or more Snellen lines and those that gained fewer than three Snellen lines (696.26 ± 234.73 µm and 648.58± 189.14 µm, respectively; p=0.46). Similarly, the CMT at the six-month follow-up did not differ between those eyes that achieved a BCVA gain of three or more Snellen lines and those that gained fewer than three (294.8 ± 60.15 µm and 336.29 ± 141.18 µm, respectively; p = 0.22).

### Intravitreal bevacizumab injections

**Figure 1:**
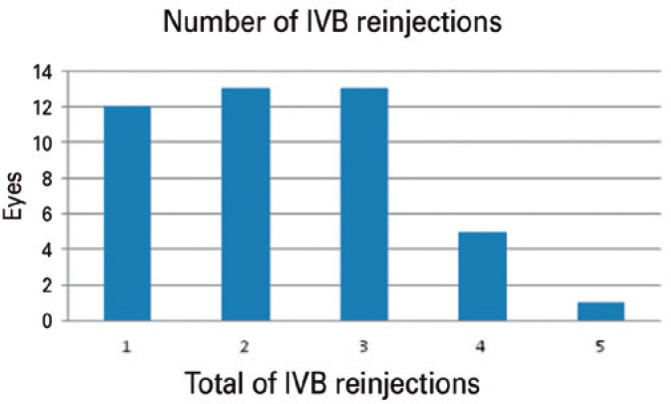
The number of intravitreal bevacizumab (IVB) reinjections required following a single dexamethasone implant in the treatment of macular edema secondary to retinal vein occlusion.

**Figure 2:**
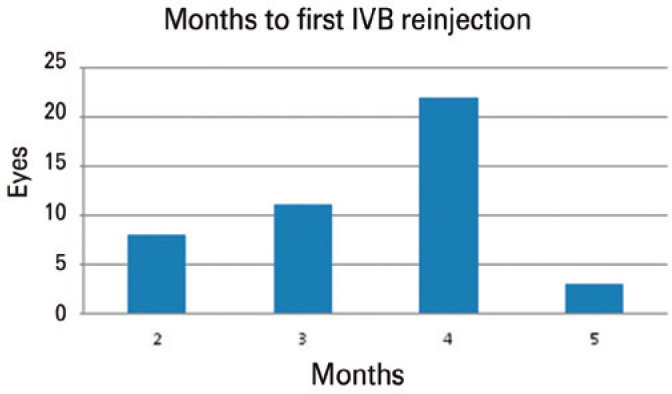
Months after dexamethasone implant when first intravitreal bevacizumab (IVB) injection was required in patients with macular edema secondary to retinal vein occlusion.

Our 44 patients were administered a total of 102 IVB injections for persistent or recurrent ME after DI. The mean number of IVB injections within the six months was 2.32 ± 1.07 (Figure 1). The mean time from DI to the first anti-VEGF injection was 3.45 ± 0.87 months (Figure 2).

## DISCUSSION

Macular edema due to RVO is a prevalent condition and its treatment is costly^(1)^. The former first-line treatment with grid photocoagulation has been supplanted by pharmacological intravitreal injections of anti-VEGF and DI^(4,6,8-10,17,27)^. Anti-VEGF drugs are widely employed due to their safety profile and effectiveness and are recommended for RVO-related ME^(4-7,10)^. The major draw­back of anti-VEGF is the need for monthly injections, especially during the first six months, to significantly increase visual acuity^(1,4,5)^. Despite an intensive therapeutic regimen, visual acuity does not improve significantly until the fourth or fifth month^(15,24)^. There seems to be no difference in the functional or anatomical results of treatment with bevacizumab, ranibizumab, or aflibercept for ME secondary to RVO^(28)^.

Conversely, DI produces an intense response during the first three months following implantation, after which, there is a wash-out effect^(9,10,15,23,29)^. Kuppermann et al. have reported a ≥15-letter improvement in BCVA as soon as seven days after DI^(30)^. A retrospective study comparing DI, ranibizumab, and aflibercept treatment of ME of RVO concluded that DI achieved the best results in terms of visual acuity by the three-month follow-up visit, with a subsequent loss of effect and the poorest functional results at six months^(29)^. This was supported by the findings of the GENEVA study group, who found that eyes treated with DI had faster recovery of visual acuity with a peak effect at two months but that effectiveness had waned by six months^(9)^. DI has been used in the treatment of other ocular conditions and is the standard of care in diabetic retinopathy^(13)^. It has been shown to delay the progression of diabetic retinopathy^(31)^, improve ME in cases refractory to anti-VEGF therapy^(32)^, and decrease re-detachment rates and proliferative vitreoretinopathy in patients who have undergone pars plana vitrectomy due to tractional retinal detachment^(33-35)^. Optical coherence tomography (OCT) allows the detection of structural abnormalities in diabetic ME such as the disorganization of retinal inner layers (DRILL) whose absence is related to a good response to DI^(36)^.

The timing of ME treatment is also an important issue. Functional improvement is known to be related to the timeliness of treatment after an ME diagnosis^(9)^. For this reason, the findings in clinical practice indicate the need for early ME treatment with DI and a shorter interval between reinjections than has previously been recommended^(37)^. DI has a good safety profile when injected once or twice^(20)^; however, there is growing concern over the side effects of glaucoma and cataracts that result from a greater number of implantations^(16,19,20)^.

Considering the sustained response to DI, and to avoid short reinjection intervals, we designed a prospective study to evaluate the effectiveness at six months of treatment-naïve ME following RVO treated with a single DI in which anti-VEGF treatment was introduced at the first sign of the washing-out effect.

This prospective study presents the anatomical and functional results of treating 44 patients with persistent or recurrent treatment-naïve ME secondary to RVO after a baseline DI with bevacizumab on an as-needed regimen.

Our study population had a mean age of 67.91 years and 54.55% were female. Of the 44 cases of RVO, 17 (38.63%) were CRVO and 27 (61.36%) were BRVO. Our study had a 20% rate of ischemic RVO. Based on prevalence studies, this is representative of RVO rates^(2,3,38)^.

The BCVA changed significantly in response to our combination therapy: 20 eyes (45.45%) improved by three or more Snellen lines by six month (44.44% of the BRVO and 47.05% of the CRVO cases). The percentage of patients who achieved a BCVA of ≥20/40 was 38.63%.

Monotherapy with DI as proposed by the GENEVA study group has been shown to achieve BCVA improvements greater than 20/40 in only 22% of eyes^(16)^. In contrast, monotherapy with anti-VEGF performed better than DI monotherapy at 24 weeks. Campochiaro et al. achieved a ≥15-letter improvement in the Early Treatment Diabetic Retinopathy Study (ETDRS) test with monthly administration of ranibizumab in more than 55% of cases of BRVO, while Brown et al. achieved improvement in 45% of eyes treated with ranibizumab^(4,5)^. A study of the clinical efficacy and safety of intravitreal aflibercept injection in patients with BRVO found that 52.7% of the eyes treated improved by at least 15 ETDRS letters at 24 weeks, with a minimum of five intravitreal aflibercept injections per eye^(8)^.

Our combination treatment regimen shows better outcomes than DI monotherapy^(16)^. However, we need to consider our study population. According to Haller, 15% of patients treated with a single dose of DI for ME due to RVO will not need additional treatment in the first year^(16)^. Our sample was composed of the 85% of patients who require more than one DI. However, our combination therapy achieved better results than DI alone and similar results to anti-VEGF monotherapy^(4,5)^. We observed a remarkably positive response in the CRVO cases compared with the BRVO cases. However, CRVO is classically considered to have worse outcomes than BRVO^(4,5)^.

There have been several recent studies of the effectiveness of combination therapy in ME of different etiologies, including diabetic retinopathy^(13,32)^. In RVO cases, these have been mainly retrospective^(18,21,23)^ and have excluded patients with ischemic RVO^(24)^ or included those previously treated with lasers or intravitreal anti-VEGF^(18,21,24)^.

Moon et al. conducted a retrospective comparison of monthly IVB administration on a *pro re nata* basis and DI followed by IVB injections in BRVO. They found faster visual recovery in the DI-treated eyes but no final functional difference between the groups at month six, with a surprisingly low number of intravitreal injections in the bevacizumab monotherapy group (2.0 ± 1.2)^(23)^. A prospective interventional case series by Singer et al. studied the effectiveness of DI treatment following bevacizumab and observed that 29% of eyes had improved by at least 15 letters at six months^(18)^. The same researchers found that treatment with DI after bevacizumab in repeated cycles increases the percentage of eyes achieving three or more lines of BCVA to 47.6%^(25)^. A prospective study with a naïve cohort compared treatment with three IVB injections followed by DI-to-DI monotherapy over six months^(22)^. They found no difference between CRVO patients from the two groups, but BRVO patients appeared to benefit more from the DI monotherapy.

There is broad agreement that the prognosis is poorer in ischemic RVO than in non-ischemic cases, with only a small proportion of eyes improving by more than two lines of BCVA^(38)^. Several studies have excluded ischemic cases for this reason^(23)^. In our study, the 20.51% of patients with ischemic RVO did not achieve significantly improved visual acuity.

CMT decreased by a mean of 352.82 ± 236.37 µm from the baseline through the follow-up. We found no differences in the baseline CMT that could predict visual acuity recovery of more than three Snellen lines nor did we find a statistical difference in CMT at six months between the patients who improved by fewer than three Snellen lines and those who improved by more than three. In conclusion, neither baseline CMT nor CMT at month six were predictive of the final BCVA.

Spectral domain-OCT provides detailed information on macular structure^(39)^. CRVO tend to have symmetrical ME while BRVO has superior or inferior ME^(40)^. Alteration to internal retinal layers and visual acuity outcomes in BRVO^(41)^ and diabetic ME have also been reported^(36,42)^. Macular thickness measurements can vary between spectral-domain and swept-source OCT. Our data should therefore be interpreted with caution when comparing them to data from swept-source OCT^(43)^.

In our study, the mean number of IVB injections was 2.32, with a mean time from DI to the first anti-VEGF injection of 3.47 months. Initiating treatment with DI and then following it up with IVB, as needed, yielded good functional results while reducing the number of injections by half. The detection of persistent or recurrent ME in our study was most effective during the second, third, and fourth months after DI, at which point 18.18%, 25%, and 50% of the patients were administered IVB.

Our study demonstrates that initiating the treatment of ME due to RVO with DI and introducing anti-VEGF promptly at the first clinical sign of steroid wash-out improves the functional and anatomical results. We achieved BCVA improvements in our sample, with better results in patients with CRVO than those with BRVO. The limitations of this study include its small sample size, short follow-up, and lack of a control group.

This combination regimen showed a synergistic increa­se in BCVA, enabling longer times between DI reinjections compared with DI monotherapy, and reducing the overall number of anti-VEGF administrations compared with anti-VEGF monotherapy^(4,5,8)^ and other combination therapies^(22)^.

In our opinion, this combined therapy is a beneficial option for patients unable or unwilling to attend monthly visits or receive monthly intravitreal injections and those patients requiring fast recovery of visual acuity. We propose a schedule of medical visits, leading to a decrease in indirect costs and patient load due to a reduced number of ophthalmology consultations. Further studies with larger cohorts comparing anti-VEGF monotherapy with our combination therapy regime are required to confirm our results.
